# Twelve months antiretroviral therapy retention among clients newly enrolled to care and treatment services in Geita Regin, Tanzania: does universal test and treat matter?

**DOI:** 10.11604/pamj.2023.46.20.40772

**Published:** 2023-09-14

**Authors:** Rachel Masuke, Yohane Kihaga, Michael Mashala, Saimon Ndalio, Omari Sukari, Onna Panga

**Affiliations:** 1 Health, Nutrition and Social Welfare Section, Geita Regional Secretariat, Geita Region, Tanzania,; 2 Management and Development for Health (MDH), Dar-es-Salaam, Tanzania

**Keywords:** Antiretroviral therapy, people living with HIV, retention, universal test and treat, Africa, HIV care

## Abstract

**Introduction:**

sustaining high rates of retention is critical for management of HIV clients, newly initiated antiretroviral therapy (ART). In low resource settings including Tanzania, retention among clients on ART was challenging due to inaccessible infrastructure, gender-based violence, inadequate skilled staff and socio-economic disparities. Low retention leads to increased morbidity and mortality. Tanzania adopted universal test and treat (UTT) strategy in mid of 2016 as recommended by Joint United Nations Program on HIV/AIDS (UNAID) that set goals for HIV epidemic control globally. Studies demonstrated controversial findings on whether UTT strategy improves retention, until now there is limited information on the effect of UTT on retaining HIV patients in our settings.

**Methods:**

a retrospective cohort study was conducted between July 2014 to June 2015 and July 2017 to June 2018 to determine 12 months ART retention among clients newly initiated ART prior and during universal test and treat (UTT) strategy in Geita Region, Tanzania. A total of 13,649 newly clients-initiated ART were extracted from the National AIDS control care and treatment database (CTC2 database). Among these clients 4,624 initiated ART prior the UTT strategy and clients 9,025 start ART after the rollout of UTT strategy. Chi-square test was deployed to determine the significant difference of proportion within categories for each UTT group. Kaplan-Meier curve and long rank test were used to determine significant differences of retention rate prior and during UTT program. Cox regression models were used to estimate the association between exposure variables and ART retention with 95% confidence intervals and p-value of p<0.05.

**Results:**

the overall mean age at ART initiation was 38 years (SD=11.6) with observed significant mean difference between two cohorts (prior UTT, mean=41, SD=11.7 Vs during UTT, mean=37, SD=11.3). The cumulative retention was 83.1% among newly initiated ART clients in both cohorts with significant difference observed between two cohorts (69.7% for prior UTT and 89.9% during UTT, p-value<0.001). The overall person year of follow up was 127,209.3 with an incidence rate of ART retention of 86 per 1000 person-year. It was significantly higher among clients enrolled during UTT strategy than clients enrolled prior UTT strategy (95.1 per 1000 PY Vs 69.6 per 1000 PY, p-value<0.001). The log rank test and Kaplan-Meier survival curve demonstrated clients enrolled in the UTT program had greater probability of retention than clients enrolled prior UTT treatment program (log rank X^2^ test = 599.2, p value < 0.001). Newly HIV clients who initiated ART after the rollout of UTT strategy had 27% higher likelihood to be retained in care and treatment as compared to clients who were enrolled prior UTT strategy, (HR=1.27; 95% CI [1.21 -1.33], p value < 0.001). Sex, District councils, World health Organisation (WHO) stage and client's visit type were significant factors associated with retention among clients newly initiated to care for both arms.

**Conclusion:**

this results, showed that probability of ART retention increased after the rollout of UTT strategy. There is a need to promote universal test and treat strategy in line with other intervention to control HIV epidemic in Geita, Tanzania.

## Introduction

Human immunodeficiency virus (HIV) epidemic is still a global public health problem with 39 million people are living with HIV globally in 2022 [1]. Sub-Saharan Africa is much affected with this epidemic largely with 67% of all people living with HIV (PLHIV) globally [2]. The number of people living with HIV increasing globally including Tanzania, however the trend of new HIV infection and AIDS related death is significantly declining [3]. The world is striving to control HIV epidemic by setting several strategies including universal test and treat (UTT) that requires all population at risk screened for HIV infection and those diagnosed HIV positive to receive early treatment regardless of their CD4 counts and WHO clinical staging contrary to previous model for ART initiation [4,5]. Tanzanian through National AIDS Control Program (NACP) has adopted UTT in 2016 aimed to control HIV infection [5]. Several intervention including integrated HIV testing services and prevention has been implemented in Tanzania through Ministry of Health in collaboration with non-governmental organization. These include index testing, Optimized providers initiative, HIV recency, focused testing, HIV self-test, uptake of pre-exposure prophylaxis and offering HIV testing at all delivery points at facility [6]. Early identification of people living with HIV and linking to care on same day regardless of CD4 count and WHO clinical staging is critically important to reduce mortality and morbidity among people living with HIV the prevalence of HIV [7]. Early detection of people living with HIV, linking them to care and retaining in ART resulted in reduced HIV advance disease progression [8]. Suboptimal retention leads to increased morbidity and mortality through various mechanisms, including suboptimal viral suppression, increased risk of drug resistance, and increased risk of HIV transmission [9]. Retention in care has been very challenging in sub-Saharan Africa. Meta-analysis in Africa showed that 80%, 77% and 72% of patients started ART remained in care after 1, 2 and 3 years on therapy respectively [10]. Universal test and treat strategy increases volume of patients initiating ART treatment leading to challenges of the ability of the facilities to absorb increased patients volume, follow and retaining them in care [11]. On the other hand, prior to the adoption of UTT strategy, all newly diagnosed people living with HIV (PLHIV) received serial individual counselling prior to treatment initiation, but retention challenges still exist [12]. The controversies exist whether UTT strategy is associated with better retention of patients into HIV care [12-14]. For instance, in Malawi patients who initiated treatment under UTT strategy had better retention compared to those who initiated prior to UTT strategy at 12 months [13]. However, in South African study also showed that compared to those who initiated treatment prior to the UTT strategy, those who initiated treatment under the UTT strategy were twice as likely to be lost at 3, 6 and 12 months [15]. Other studies reported several factors related to loss to follow up such as distance to the health facility, poverty, lack of knowledge, misperceptions of treatment, stigma, lack of social support, seeking care from alternative healing system, CD4 count, age, pregnancy and breastfeeding [13-16]. The adoption of UTT strategy, may have additional retention challenges and its evaluation has not been studied in our setting. Therefore, our study determined the effect of UTT strategy on ART retention among HIV clients in Geita Region.

## Methods

**Study design and setting:** a retrospective cohort study was conducted to determine ART retention among the newly initiated clients prior and during the adoption of UTT strategy in Geita Region. Geita is one of the 26 Tanzania Mainland Region located in the lake zone with an estimated total population of 2.9 million with average growth rate of 5.4 per annual [17]. Administratively, the region has 5 districts with 6 councils namely Bukombe DC, Chato DC, Geita DC, Geita TC, Mbogwe DC and Nyang´hwale DC. The main economic activities in the region include mining, agriculture and fisheries [18]. The total number of 218 public and private health facilities providing primary and secondary health care services and of these 103 facilities provide care and treatment clinics in the region.

**Study population and period:** the study was conducted among adults both male and female of 15 years or older, newly diagnosed with HIV and enrolled to care and treatment between July 2013 to June 2014 (Prior UTT strategy) and July 2017 to June 2018 (during UTT strategy). Clients who newly diagnosed are emotionally and psychologically unstable and had likelihood of suboptimal retention to care [19]. We excluded clients with missing outcome and less than 15 years old.

**Sample size estimation and power calculation:** the study utilizes adults aged 15+ and who are newly diagnosed with HIV and enrolled to care and treatment at different times. Power was calculated using Cox proportion hazards model by STATA statistical package. Considering assumptions like HZ=1.27, 51% in UTT clients, 5% marginal error, and sample size of 13649; Stata command; *power Cox, n (13649), Hazard ratio* (1.27), *effect (hratio)*, resulted to a study power of above 90%.

**Data sources:** data was extracted from National AIDS control electronic care and treatment database (CTC2). The database includes sociodemographic (age, sex, marital status, residence, facility tiers), clinical information (CD4 count, WHO clinical staging), others (date of ART initiation, Visit date, ART regime, clients type visit, client´s category, appointment date). Data completeness and accuracy was assured by checking gaps and values out of range and gaps identified were recited to ensure validity and reliability.

**Consent procedure:** permission to conduct this study was obtained from the Regional Commissioner´s Office. To keep confidentiality, participant´s names were not included, rather CTC number was used to identify the participants.

### Variables and definitions

**Retention on antiretroviral therapy:** was defined as the number of clients who were on ART 12 months prior and are still on ART at the end of the study period. Clients can access ART at either facility visits, outreach services or decentralized drug dispensing services for 12 months (20).

**Loss to follow up:** was defined as the number of clients with no clinical contact for 28 days after the last scheduled appointment or expected clinical contact (20).

**Missing appointment:** clients who missed clinics visit from 3 to 28 days (20).

**Transferred out:** patient was confirmed to be successfully transferred to another health facility during the reporting period [20].

**Opted out:** patient was contacted and confirmed to have stopped ART during this reporting period (20).

### Independent variables

**Primary exposure:** mode of ART delivery: clients who initiated ART from July 2013 to June 2014 were categorized as prior to the adoption of UTT strategy, and clients-initiated ART from July 2017 to June 2018 were categorized as UTT strategy.

**Secondary independent variables:** age, sex, marital status, residence/councils, ART start date, level of health care facility, type of regimen, WHO clinical stage, ART categories and client categories, time to linkage (number of days from HIV positive diagnosis to ART initiation) and client visit types.

**Data management and analysis:** data was extracted from CTC2 database and then exported to STATA version 14 for cleaning and analysis. Categorical variables were summarized using frequency distributions while continuous variables were summarized using mean and standard deviation (SD) or median and interquartile range (IQR). Survival analysis was used to estimate probability of ART retention among HIV clients prior and during the adoption of UTT. Kaplan-Meier survival curve was used to determine the median time to retention while log rank test was used to determine the difference ART retention prior and during UTT program. Univariate Cox regression model was used to estimate the association between exposure and outcome variables. A multivariable model was constructed by variables that had a p-value of less than 0.1 in univariate analysis. Likelihood ratios (or AIC) were used to obtain the final model. Being our key exposure, the mode of ART delivery was included in the final model regardless of its p-value. A 95% confidence interval was used to assess the strength of associations and a p-value less than 0.05 was set as a significant level.

## Results

**Figure 1:**
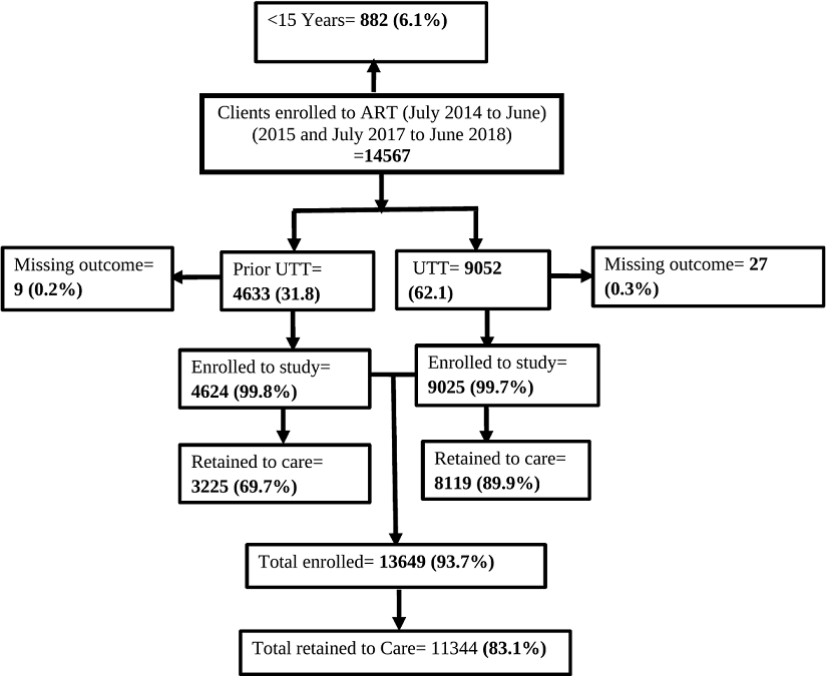
flow chart for selected people living with HIV clients included in the study

A total of 14,567 clients were extracted from the electronic CTC database from 103 health facilities in the Geita Region. We excluded 36 clients due to missing outcome status and 882 children age below 15 years. Thirteen thousand, six thousand and six hundred forty-nine (13,649) were included in the study. Among these, 4,624 (33.9%) enrolled to ART prior to the rollout of the Universal test and treat strategy and 9,025 clients (66.1%) enrolled to ART during the UTT strategy (Figure 1). The overall mean age at ART enrollment was 38 (SD=11.6) with the significant mean age difference between the two groups, prior (mean= 41, SD=11.7) and at UTT program (37 years, SD=11.3) p-value<0.001. The median time from diagnosis to the initiation of ART was 1 day (IQR= 0-15), clients enrolled prior the UTT period took longer time to start ART from HIV diagnosis as compared to clients enrolled during UTT strategy. Nearly two third (65.3%) of the clients enrolled in this study were Female, residing in Geita District council (35.1) and 63.5% were either married or cohabiting (Table 1). During the ART initiation, 34.5% of clients were in WHO stage III, Majority of HIV clients (99%) were on first line of treatment regimen and more than two thirds of the participants during UTT were enrolled to ART less 7 days while prior UTT only 37.6% enrolled to care, this figure is statistically difference with p-value<0.001 (Table 2).

**Table 1:** socio-demographic and clinical information of clients in this study

Variables	Number	Percent
Mean age (in years)	38.4	SD = 11.6
Time to linkage (in days)	1	IQR= 0-15
**Age at start of ART**		
15-24	1,332	9.8
25-34	4,670	34.2
35-50	5,559	40.7
50+	2,088	15.3
**Sex**		
Female	8,910	65.3
Male	4,739	34.7
**Council**		
Bukombe DC	1,774	13.0
Chato DC	3,018	22.1
Geita DC	4,786	35.1
Geita TC	1,689	12.4
Mbogwe DC	1,611	11.8
Nyang'hwale DC	771	5.7
**Marital status**		
Single	2,276	16.8
Divorced	2,021	14.8
Married/cohabiting	8,667	63.5
widowed	685	5.0
**Visit type code**		
Unscheduled	10,584	77.5
Scheduled	3,065	22.5
**WHO stage**		
Stage I	4,192	30.7
Stage II	3,326	24.4
Stage III	4,704	34.5
Stage IV	1,427	10.5
**Treatment regimes**		
First line	13,604	99.7
Second line	45	0.3
**Time to linkage**		
<= 7 days	8,974	65.7
>7 days	4,675	34.3
**Retention on ART**		
Retained	11,344	83.1
Not retained	2,305	16.9

ART**:** antiretroviral therapy; SD: standard deviation; IQR: interquartile range; WHO: World Health Organisation

**Table 2:** clinical and sociodemographic characteristics of study participants by services delivery program (prior and during universal test and treat strategy)

Variables	Prior-UTT, n (%)	During-UTT, n (%)	P-value
Mean age	41 (SD = 11.7)	37 (SD = 11.3)	
Time to linkage	15 (IQR= 1 - 77)	0 (IQR=0 - 5)	
**Age at start of ART**			
15-24	216 (16.2)	1,116 (83.8)	<0.001
25-34	1,298 (27.8)	3,372 (72.2)	
35-50	2,183 (39.3)	3,376 (60.7)	
50+	927 (44.4)	1,161 (55.6)	
**Sex**			0.986
Female	3,019 (65.3)	5,891 (65.3)	
Male	1,605 (34.7)	3,134 (34.7)	
**Council**			<0.001
Bukombe DC	818 (17.7)	956 (10.6)	
Chato DC	892 (19.3)	2,126 (23.6)	
Geita DC	1,224 (26.5)	3,562 (39.5)	
Geita TC	673 (14.5)	1,016 (11.3)	
Mbogwe DC	650 (14.1)	961 (10.6)	
Nyang'hwale DC	367 (7.9)	404 (4.5)	
**Marital status**			<0.001
Single	716 (15.5)	1,560 (17.3)	
Divorced	759 (16.4)	1,262 (14.0)	
Married/cohabiting	2,843 (61.5)	5,824 (64.5)	
widowed	306 (6.6)	379 (4.2)	
**Visit type code**			<0.001
Unscheduled	3,920 (84.8)	6,664 (73.8)	
Scheduled	704 (15.2)	2,361 (26.2)	
**WHO stage**			<0.001
Stage I	788 (17.0)	3,404 (37.7)	
Stage II	747 (16.2)	2,579 (28.6)	
Stage III	2,150 (46.5)	2,554 (28.3)	
Stage IV	939 (20.3)	488 (5.4)	
**Treatment regimes**			<0.001
First line	4,590 (99.3)	9,014 (99.9)	
Second line	34 (0.7)	11 (0.1)	
**Time to linkage**			<0.001
<= 7 days	1,737 (37.6)	7,237 (80.2)	
>7 days	2,887 (62.4)	1,788 (19.8)	
**Retention on ART**			<0.001
Retained	3,225 (69.7)	8,119 (90.0)	
Not retained	1,339 (30.3)	906 (10.0)	

ART**:** antiretroviral therapy; UTT: universal test and treat strategy; WHO: World Health Organisation

**Proportion of antiretroviral therapy retention:** the overall proportion of ART retention among clients newly initiated ART was 83.1%. The proportion of ART retention in UTT was higher (89.9%) as compared to clients initiated prior UTT (69.7%), this difference was statistically difference with p-value <0. 001 (Figure 1).

**Figure 2:**
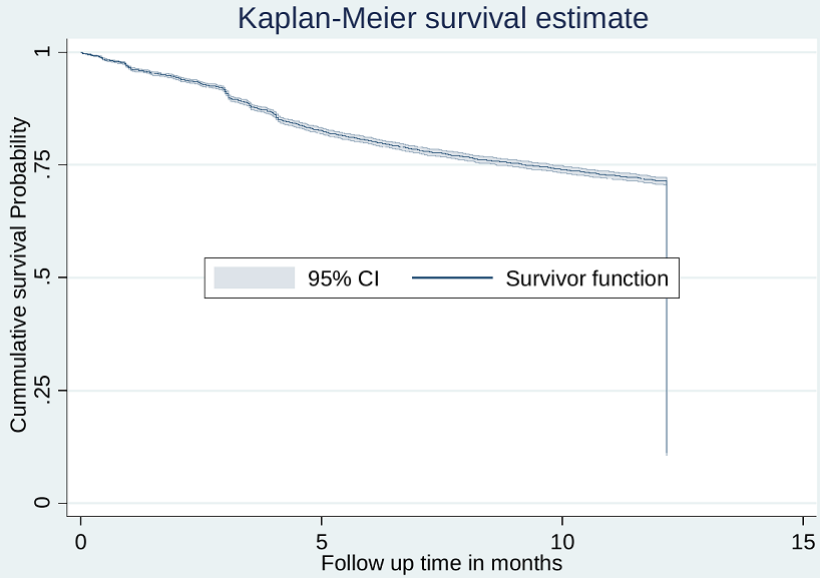
cumulative survival estimate for antiretroviral therapy retention among people living with HIV initiated antiretroviral therapy in Geita Region (July 2014-June 2015 and July 2017-June 2018)

**Figure 3:**
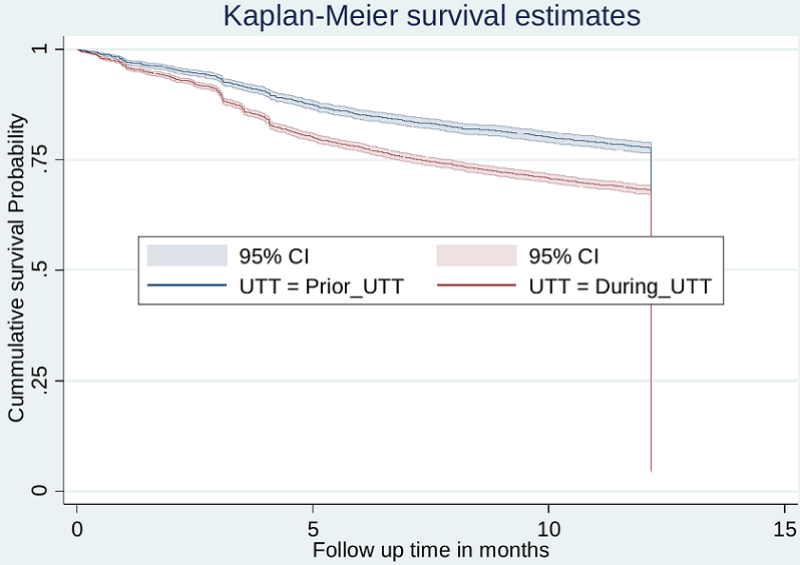
Kaplan-Meier survival estimate for antiretroviral therapy retention among people living with HIV initiated antiretroviral therapy prior universal test and treat Vs during universal test and treat strategy in Geita Region

**Rate of antiretroviral therapy retention:** a total of 13,649 newly enrolled to care, and treatment services were followed for different period of time making overall 127,209.3-person year of observation. The overall incidence rate of ART retention was 86 per 1000 person-year (95% CI: (84.4 - 87.6). The incidence rate of ART retention was higher among clients enrolled during UTT strategy (IR = 95.1; 95% CI: 93.1 - 97.3) as compared to clients enrolled prior UTT strategy (IR = 69.6; 95%CI: 67.2 - 72.1) per 1000 person-year of observation. The median survival time was undetermined because the largest observed analysis time was censored; the survivor function does not go to zero (Figure 2). The probability of survival was almost the same for the first three months for clients in both arms. The log rank test and Kaplan-Meier survival curve demonstrated clients enrolled in the UTT program had greater probability of retention than clients enrolled prior UTT treatment program (log rank X^2^test = 599.2, p value < 0.001) (Figure 3).

**Factors associated with antiretroviral therapy retention (univariable and multivariable Cox regression analysis):** in the univariable Cox regression, program of enrolment (Prior UTT Vs during UTT), District council, age categories, sex, treatment regimes, WHO stage, time to linkage to care and visit type were associated with ART Retention. By using variables which have p value less than 0.10 in the bivariate analysis, multivariable Cox regression was fitted with forward stepwise method. After controlling the effect of other variables, residence/district councils, program at enrolment, sex, time to linkage to care, WHO clinical stage and visit type were significant predictors of ART retention among clients enrolled to care and treatment services. After controlling the effect of other variables, the likelihood of ART Retention was 27% higher for clients enrolled during universal test and treat strategy than the patients who were enrolled prior UTT strategy rollout (aHR: 1.27.95% CI: 1.21 - 1.34, p value < 0.001). In addition, the probability of ART retention was 11% higher among clients residing in Chato DC, 19% higher among clients residing in Geita DC, 19% higher among clients in residing in Geita TC, 15% higher among clients in residing in Mbogwe DC and 8% higher among clients in residing in Nyang´hwale DC as compared to clients residing in Bukombe DC. Male client had 8% higher chance of being retained in care as compared to female clients (aHR: 1.08, 95% CI: 1.04 - 1.13, p value < 0.001). Also, client with scheduled visit had 58% higher chance of being retained on care as compared to client with unscheduled visit, aHR 1.58, 95% CI: 1.51 - 1.66, p value < 0.001. On the other hand the likelihood of Retention was 9%, 17% and 2% lower among clients in WHO stage II, III and IV respectively as compared to clients in WHO stage one, (aHR=0.91, 95%CI: 0.87 - 0.96, p value<0.001; aHR=0.83, 95% CI: 0.79 - 0.87, p value < 0.001 and aHR=0.98, 95% CI: 0.91 - 1.05, p value<0.00) respectively. Client those takes more than 7 days to start ART from the date of HIV positive diagnosis had 8% lower hazard of ART retention as compared to client linked to care within 7 days from the date of HIV positive diagnosis, aHR=0.92, 95% CI: 1.88 - 0.97, p value< 0.001 (Table 3).

**Table 3:** Cox regression analysis for factors associated with antiretroviral therapy retention among HIV clients newly initiated antiretroviral therapy

Variables	Univariable cox regression analysis			Multivariable cox regression analysis		
	Haz ratio	95% conf interval	P-value	Haz ratio	95% conf interval	P-value
**Treatment program**						
Prior UTT	1			1		
During UTT	1.40	1.35 - 1.46	<0.001	1.27	1.21 - 1.34	<0.001
**Age categories (in years)**						
15 - 24	1			1		
25 - 34	0.92	0.86 - 0.99	0.023	0.97	0.90 - 1.04	0.314
35 - 50	0.84	0.79 - 0.90	<0.001	0.93	0.87 - 1.00	0.039
50 and above	0.83	0.77 - 0.90	<0.001	0.94	0.86 - 1.02	0.122
**Council**						
Bukombe Dc	1			1		
Chato Dc	1.15	1.07 - 1.23	<0.001	1.11	1.03 - 1.18	0.005
Geita Dc	1.20	1.13 - 1.28	<0.001	1.19	1.12 - 1.27	<0.001
Geita Tc	1.15	1.06 - 1.24	<0.001	1.19	1.10 - 1.29	<0.001
Mbogwe Dc	1.11	1.03 - 1.20	0.009	1.15	1.07 - 1.25	<0.001
Nyang'hwale Dc	1.07	0.97 - 1.18	0.165	1.08	0.98 - 1.18	0.118
**Sex**						
Female	1			1		
Male	1.05	1.01 - 1.09	0.019	1.08	1.04 - 1.13	<0.001
**WHO stage**						
Stage I	1			1		
Stage II	0.88	0.83 - 0.92	<0.001	0.91	0.87 - 0.96	0.001
Stage III	0.75	0.72 - 0.79	<0.001	0.83	0.79 - 0.87	<0.001
Stage IV	0.83	0.78 - 0.89	<0.001	0.98	0.91 - 1.05	0.602
**Treatment regimes**						
First line	1			1		
Second line	0.81	0.59 - 0.90	0.01	0.97	0.71 - 1.34	0.863
**Time to linkage**						
<7 days	1			1		
≥7 days	0.80	0.77 - 0.83	<0.001	0.92	0.88 - 0.97	0.001
**Visit type code**						
Unscheduled	1			1		
Scheduled	1.63	1.55 - 1.70	<0.001	1.58	1.51 - 1.66	<0.001

ART**:** antiretroviral therapy; UTT: universal test and treat strategy; WHO: World Health Organization

## Discussion

This study assessed the effect of UTT strategy on ART retention among clients newly initiated ART in Geita Region, Tanzania. The overall proportion of ART retention among newly initiated clients was 83.1% with significant difference observed between Prior UTT (69.7%) and during UTT (89.9%), p-value <0.001. After adjusting other variables, factors significantly associated to ART retention was UTT program, residence, sex, WHO clinical staging and visits type. Our study reported overall annual ART retention of 83.1% among clients enrolled in care and Treatment services, which differ significantly between the two cohorts (Prior, 69.7% and at UTT program, 89.9%). However, this finding is lower than the set target of UNIADS first 95% [4]. Also, this figure is higher than study done in Malawi and South Ethiopia that reported 74.7% and 63% respectively [13,21]. This difference is explained by the fact that the care and treatment services is fully supported by Non-Government Organization (NGO) under United States President´s emergency plan for AIDS relief through center for disease and control prevention. During the UTT program, health care providers, expert clients and community based HIV services volunteers were deployed at each health facility that offering Care and Treatment Center to ensure clients tracking, retaining to care, and updating client´s information. The rate of ART Retention was higher for patients enrolled during UTT strategy than the patients who were enrolled prior to the UTT strategy rollout. This results were similar to study done in Malawi documented 16% ART retention higher in UTT versus prior UTT and another study in Uganda found an increase in retention by 9.2% after the scale up of UTT [13,22]. This evidence suggested that initiating ART earlier could reduce HIV incidence, increase ART coverage and retention to care [4]. Similarly, there are potential benefits of universal test and treat strategy on effective linkage to and retention in HIV clients in care and treatment clinics [16]. A study done in South Africa reported contrast finding that demonstrated clients enrolled at UTT program had twice more to be lost as compared to those initiated prior UTT [14]. This highlight that this study done at early adoption of UTT where differentiated services delivery model was not launched at South Africa [23]. Universal test and treat increases volume of patients initiating ART treatment hence this strategy faced the challenge of the ability of the facilities to absorb increased patients volume, follow and retaining them in care [22]. Our study found that clients that take more than 7 days to start ART from the date of HIV positive diagnosis had 8% lower hazard of ART retention as compared to client that linked to care within 7 days from the date of HIV positive diagnosis. This study supports the practice of offering same-day ART initiation after HIV positive testing. This result align with the finding from Lesotho which indicate higher rates of linkage to care to patient initiate ART on the same day as compared to usual care [12].

## Conclusion

Retention on ART among newly HIV clients was 83.1%. This result, highlight the need for targeted interventions for these groups to achieve the 95-95-95 UNAIDS targets in the UTT era. The rate of ART Retention was higher for patients enrolled during universal test and treatment strategy than the patients who were enrolled prior UTT strategy rollout. Our study had some limitations, including missing data on CD4 count and viral load results. The latter two variables could therefore not be included in the analysis. For that case, the study was not able to address all reasons affecting retention in care.

### What is known about this topic

- *The Joint United Nations Programme on HIV and AIDS targets stipulate that 95% of all people receiving antiretroviral therapy should be retained in care and have durable viral suppression*.

### What this study adds

- *Estimates of antiretroviral therapy retention rate in patients initiated antiretroviral therapy through the prior-universal test and treat strategy and during universal test and treat strategies;*
- *The likelihood of antiretroviral therapy retention was higher for patients enrolled during universal test and treat strategy than the patients who were enrolled prior to the universal test and treat strategy rollout;*
- *Universal test and treat strategy improves antiretroviral therapy retention for clients newly initiated to care and treatment*.
